# The association between dietary consumption, anthropometric measures and body composition of rural and urban Ghanaian adults: a comparative cross-sectional study

**DOI:** 10.1186/s40795-020-00339-6

**Published:** 2020-05-25

**Authors:** Nana Ama F. Agyapong, Reginald A. Annan, Charles Apprey, Linda N. E. Aduku, Elizabeth C. Swart

**Affiliations:** 1 Department of Biochemistry and Biotechnology, Kwame Nkrumah University of Science and Technology, Kumasi, Ghana; 2 Department of Dietetics and Nutrition, University of the Western Cape, Cape Town, South Africa

**Keywords:** Overweight, Obesity, Body composition, Waist circumference, Visceral fat, Body mass index

## Abstract

**Background:**

Overweight and obesity have become threats to public health in all regions across the globe including sub-Saharan Africa where prevalence used to be low. Policies to regulate the food environment and promote healthy food consumption look promising to reducing the prevalence of obesity but in Ghana there is not enough data to elicit a policy response. This study assessed the association between dietary consumption, anthropometric measures, body composition and physical activity among rural and urban Ghanaian adults.

**Methods:**

This was a cross-sectional study involving 565 Ghanaian adults. Structured interviewer administered questionnaires were used to collect information on socio-demographics. Dietary consumption was assessed using household food frequency questionnaire and 24-h recall. Height, weight, BMI, waist circumference and body composition of all participants were determined. The World Health Organization’s Global Physical Activity Questionnaire (GPAQ) was used to assess physical activity levels. Mann-Whitney U test was used to analyze differences in anthropometric measures, body composition and consumption among rural and urban participants. Principal component analysis was used to analyze household food frequency data and nutrient analysis template was used to analyze 24-h recall. Chi-square was used to measure differences in obesity prevalence by community and gender. Multinomial logistic regression was used to model the risk factors associated with obesity.

**Results:**

The prevalence of overweight and obesity using BMI were 29.9 and 22.9% respectively. Use of waist circumference measurement resulted in the highest overall obesity prevalence of 41.5%. Prevalence of obesity was higher among females compared to males across all measures with the exception of visceral fat that showed no significant difference. Four different patterns were derived from principal component analysis. Among urban participants, the staple pattern showed a significant negative correlation with visceral fat (r − 0.186, *p*-value 0.013) and BMI (r − 0.163, *p*-value 0.029). Multinomial logistic regression revealed that males (AOR 19.715, CI 9.723–39.978, p-value < 0.001) had higher odds of being of normal weight compared to females.

**Conclusion:**

Prevalence of overweight and obesity continue to rise in Ghana, especially among females. Public education and screening as well as interventions that regulate the food environment and make affordable and available healthy food options are needed to control the rise in obesity prevalence.

## Background

Obesity is a major cause of cardiovascular and chronic diseases which contribute to about 71% of all global deaths [1]. Obesity continues to increase across all ages and regions all over the world [2]. In sub-Saharan Africa where about 42% of the population live below the poverty line, prevalence of obesity continues to rise although a strong positive association has consistently been observed between socio-economic status and obesity [3]. Annually, about 2.8 million people die from overweight or obesity [1]. Once a problem of the developed world, obesity is now on the increase in developing countries and Ghana is no different. It is forecasted that by 2030, obesity will be more prevalent in developing countries [4] and Dake [5] projects prevalence among Ghanaian women aged 15–49 years to reach 15.1% by 2023. Developing countries are faced with a double burden of disease not only at country and community levels but also within households. This implies that in a household where adults are obese, it is possible to find undernourished children who struggle to achieve optimum growth z-scores for their age [1]. Stunting and wasting are risk factors for obesity in later years and this situation creates a cycle of malnutrition in the sub-region [6].

Genetic susceptibility, high socio-economic status, excess caloric consumption among other factors predict obesity but the current transition and trend is mainly driven by increased caloric intake and reduced physical activity levels that are associated with urbanization coupled with lack of policies to control an obesogenic food environment [7, 8]. Urbanization leads to reduced consumption of healthy staples and an obesogenic food environment makes readily available cheaper priced energy dense foods [9]. Energy balance is key to the maintenance of body weight and consumption of more calories than what is utilized through metabolic and physical activities leads to weight gain. These excess calories, stored in the body as fat can impair proper health and function [10]. Additionally, inadequate intakes and deficiencies of micronutrients such as zinc are linked to obesity [11].

Consistently, studies have reported a higher prevalence of obesity among urban populations compared to rural ones. Part of this may be due to differences in physical activity levels, socio-economic status and food consumption patterns among rural and urban dwellers [12, 13]. Gender differences have also been observed with females been more susceptible than males. Parity is one factor that puts women at higher risk than men [14, 15].

Body mass index is the usual screening tool for determining obesity among populations [15]. Build-up of body fat positively correlates with total body mass and hence weight gain indices are used as determinants of body fat [16]. It however, has limitations of not been able to accurately predict adiposity [17]. BMI, when used in combination with other diagnostic tools such as waist circumference and Body Impedance Analysis (BIA) provides in-depth information about adiposity as well as presumed cardio-metabolic risk. Increased body fat is a prominent risk factor for type 2 diabetes, stroke, hypertension and heart disease [18, 19]. Combining these tools to screen for obesity among populations may reveal higher prevalence than what is reported by most studies.

The Ghana 2014 Demographic Survey (GDHS) indicates that the prevalence of overweight and obesity among men and women is 15.7 and 40.1% respectively [20]. Some recent studies have been conducted within various Ghanaian populations to ascertain the prevalence of overweight and obesity but these studies have mainly focused on urban females and few have been community based. Few studies have compared rural and urban individuals as well as included body composition measurements. Additionally, some studies have assessed how single food items and nutrients predict overweight and obesity but no study exists in Ghana that has determined the influence of broad consumption patterns on obesity. Assessment of all these parameters will provide more insight in to the obesity menace and consumption patterns associated with it. Consumption patterns are necessary to formulate policies that support the selection and consumption of healthier food choices as a means to curb the peril of obesity.

This study is part of a larger study titled “Researching the Obesogenic Food Environment, its Drivers and Potential Policy levers in Ghana and South Africa (ROFE). The project consists of three phases and the findings presented in this paper are from phase one of the project. The main aim of this study was to compare the prevalence of obesity among rural and urban dwellers using BMI, waist circumference and body composition measures and to determine consumption factors associated with obesity.

## Methods

This was a cross-sectional study that involved the use of interviewer-administered structured questionnaires to collect information on socio-demographics. The questionnaire was pretested prior to data collection. Sociodemographic data collected included age, sex and level of education. Weight, height, waist circumference and body composition measurements of all participants were taken. The height of each participant was entered in to the body composition analyzer which was used to measure the weight and other body components. Body Mass Index (BMI) was generated from the weight and height measurement (weight in kilograms/ height in metres^2^).

### Study site

The study was conducted in two communities in the Ashanti region of Ghana; Ahodwo, an urban community and Ejuratia, a rural community. Data collection was done by visiting households within the selected communities.

### Sample size and sampling

A total of 600 individuals participated in this study. The sample size of 600, 300 from rural and 300 from urban was determined by the ROFE research team to be adequate for the study. Out of this number, 35 did not complete anthropometric and body composition measurement and were omitted from this analysis. This paper therefore, reports data on 565 participants. A Systematic sampling was used to select households and any household member either male or female eighteen years and above was included. One household was randomly selected within each house. For each household, only one member was included and where there were both a male and a female who qualified, the male was selected to ensure gender balance as it was more difficult to find males in households. Additionally, the first adult seen in the household was the one selected to be part of the study unless he or she refused and another was selected. In the urban area, the main streets of the town were used to divide the locality into six parts. After a random start at each main street, every fifth household was selected to form part of the study. In the rural community, the main station was used as the central point to divide the town into four parts and after a random start at each point, every third household was selected. The difference in intervals was due to the smaller sample frame in the rural area compared to the urban area. Households with no adult member present and households who declined to participate in the study were excluded.

### Dietary consumption

Twenty four-hour recall and household food frequency was used to collect information on food consumption. The household food frequency consisted of commonly consumed food groups. Participants were also asked of any likely foods under each of the food groups they had consumed that were not captioned. Participants were to indicate how often they consumed each food item. One twenty-four-hour recall was taken for each participant to assess previous day food consumption. Principal component analysis was used to generate patterns of consumption among participants. Nutrient analysis template; a food composition table consisting of only Ghanaian foods was used to estimate quantity of nutrient intakes and dietary diversity from the 24-h recall.

### Body composition and anthropometric measurement

Participants were made to remove their footwear and put on light clothing prior to the taking of anthropometry. Height was taken using Seca stadiometre, model 213 with participants standing up right with feet together and hands at the sides. Weight and body composition were measured using the Omron body composition monitor, model HBF-514. Height, gender and age of participants were entered into the body composition monitor before participants were made to stand on it. This generated body composition results as well as BMI of participants. Body mass index (kg/m^2^) was classified using WHO criteria for adults; < 18.5 underweight, 18.5–24.9 normal, 25–29.9 overweight and ≥ 30 obese. Visceral fat of > 9% was definitive of central obesity and body fat cut-offs were based on gender and age of participants as suggested by Gallager [19, 21]. Waist circumference was taken with a flexible tape measure. Central obesity was defined as waist circumference of > 88 cm for females and > 102 cm for males.

### Assessment of physical activity

The World Health Organization’s Global Physical Activity Questionnaire (GPAQ) was used to assess the physical activity levels of study participants. The total minutes of different level of physical activities performed by participants in a typical week was calculated from the number of days in a week for engaging in such activity and the time spent on the particular activity. The activity ranged from moderate to vigorous intensity activity or sports as well as walking and bicycling as a means of travelling. Total time for vigorous intensity activities for the week was multiplied by 8 while moderate intensity activities, walking and bicycling were multiplied by 4 to convert the minutes to metabolic equivalents. The World Health Organization (WHO) suggests a minimum of 600 metabolic equivalents per week for adults as a way to promote cardiometabolic health. Participants were stratified in to two groups based on those meeting the recommendation and those not meeting the recommendation.

### Ethical clearance

Ethical clearance for the study was granted by the Council for Scientific and Industrial Research (CSIR), Ghana; RPN 011/CSIR-IRB/2017. Written permission was sought from local government officials before data collection and all participants signed or thumb printed an inform consent form to indicate voluntary participation.

### Statistical analysis

Statistical Package for Social Sciences (IBM SPSS) 23 was used for data analysis. Normality test revealed that data was positively skewed. Mann-Whitney U test was used to compare the median age, body composition and nutritional intakes among rural and urban participants. Chi-Square was used to determine the difference in obesity prevalence and physical activity levels among males and females as well as among rural and urban dwellers. In instances where cell counts were less than five, Fisher Exact Test was used. Spearman correlation was used to determine the relationship between BMI and body composition measures. Principal component analysis was used to determine the patterns of consumption. Multinomial logistic regression was used to determine the predictors of obesity measured by waist circumference. Waist circumference was used because it determined the highest prevalence of obesity and strongly correlated positively with all other body composition measures. A *p*-value of < 0.05 was set as statistically significant.

## Results

A total of 565 participants took part in the study; 292 from rural and 272 from urban. The median age of participants was 40(26) years and there was no statistical difference between rural and urban participants. The overall median body mass index of participants was 25.6(7.4) with urban dwellers recording a significant higher value. Visceral fat was also significantly higher for urban dwellers compared to rural participants. Calorie, carbohydrate and fibre intakes were significantly higher among rural participants while urban participant consumed higher vitamin A. Other nutrients did not show any significant difference. Rural participants had higher metabolic equivalents compared to their urban counterparts. Table 1 shows socio-demographics, nutritional intake and body composition characteristics of study participants.

**Table 1:** Descriptive Statistics

DESCRIPTIVE VARIABLES	Total *N* = 565	STATISTICS AND BALANCE CHECK		
		RURAL *n* = 292	URBAN *n* = 272	*P*-VALUE
		n(%)or Median (IQR)	n(%)or Median (IQR)	
Age	40 (26)	40 (26)	38 (26)	**0.737**
Gender				
Male	113 (19.8)	43 (14.7)	70 (25.7)	0.001*
Female	452 (79.2)	249 (85.3)	202 (74.3)	
Level of Education				
No formal education	98 (17.2)	60 (21.4)	38 (16.7)	< 0.001*
Primary	50 (8.8)	27 (9.6)	23 (10.1)	
Junior secondary	176 (30.8)	120 (42.9)	56 (24.7)	
Senior secondary	132 (23.1)	64 (22.9)	66 (29.1)	
Tertiary	53 (9.3)	9 (3.2)	44 (19.4)	
Weight (kg)	65.9 (20.2)	62.4 (18.3)	69.9 (20.65)	< 0.001*
Height (cm)	159.3 (9.83)	158.0 (8.60)	160.5 (11.2)	< 0.001*
BMI (kg/m^2^)	25.6 (7.40)	25.0 (6.7)	26.1 (7.65)	0.016*
Waist circumference (cm)	87.4 (19.80)	87.0 (17.55)	89.0 (22.0)	**0.115**
Muscle mass	26.9 (6.13)	26.8 (5.05)	27.0 (6.95)	**0.526**
Body fat	35.7 (15.22)	35.2 (14.33)	36.9 (16.35)	**0.341**
Visceral fat	7.0 (4.0)	7.0 (4.0))	7.0 (5.0)	0.015*
Energy intake (Kcal)	1434.0 (951.1)	1601.4 (999.04)	1330.7 (899.84)	0.004*
Fat (g)	42.5 (44.08)	44.2 (50.8)	41.0 (39.2)	**0.781**
Protein (g)	42.6 (32.5)	42.6 (32.7)	42.9 (33.06)	**0.838**
Carbohydrate (g)	208.0((137.0)	223.5 (128.8)	187.9 (137.6)	0.001*
Fibre (g)	18.3 (13.3)	20.0 (13.8)	16.9 (13.5)	0.001*
Sugar (g)	29.6 (41.4)	28.0 (35.6)	33.9 (50.04)	**0.242**
Folate (μg)	225.6 (240)	234 (241.2)	216.4 (237.0)	**0.222**
Iron (mg)	9.2 (6.6)	9.5 (6.33)	8.8 (6.8)	**0.569**
Zinc (mg)	5.92 (4.7)	6.1 (4.9)	5.7 (4.7)	**0.860**
Vitamin B_12_ (μg)	1.76 (3.2)	1.92 (3.81)	1.73 (2.64)	**0.296**
Vitamin A (μg)	120.6 (139.8)	111.2 (121.6)	128.8 (165.3)	0.022*
Vitamin E (mg)	5.6 (6.4)	5.6 (6.4)	5.5 (6.3)	**0.658**
Saturated fat (g)	12.2 (15.3)	11.1 (15.8)	13.1 (14.6)	**0.143**
Monounsaturated fat (g)	14.7 (18.3)	14.8 (20.33)	14.7 (17.0)	**0.930**
Polyunsaturated fat (g)	7.9 (8.4)	8.37 (9.17)	7.3 (7.7)	**0.285**
Dietary diversity score	6.0 (2.01)	6 (3)	6 (2.01)	**0.118**
Physical activity (total metabolic equivalent per week)	600 (3280)	960 (4360)	360 (1890)	< 0.001*

Some variable responses were missing and therefore does not sum up to 565. Chi-square was used to analyze categorical variable. Mann Whitney U test was used to analyze continuous variable. *Significant at *P*-value < 0.05

Using BMI, there was no difference in the prevalence of obesity among rural and urban participants. Visceral and body fat cut-offs showed a higher prevalence of obesity in urban compared to rural participants. Prevalence of obesity across all parameters was higher among females compared to males with the exception of visceral fat that showed no difference. Table 2 shows the prevalence of obesity by community and gender.

**Table 2:** Prevalence of obesity stratified by community and gender

Variable	Total	Rural *n* = 292	Urban *n* = 272	*p*-value	Male *n* = 113	Female *n* = 452	*P*-value
BMI							
Underweight	9 (1.6)	5 (1.7)	4 (1.5)	0.105	0 (0)	9 (2.0)	
Normal	247 (43.3)	140 (48.3)	104 (39.4)		74 (67.3)	171 (38.4)	< 0.001*
Overweight	171 (29.9)	87 (30.0)	83 (31.4)		25 (25.7)	145 (32.6)	
Obese	131 (22.9)	58 (20.0)	73 (27.7)		11 (10.0)	120 (27.0)	
Visceral fat							
Normal	434 (76.0)	246 (85.1)	184 (71.0)	< 0.001*	84 (77.1)	347 (78.9)	0.385
Obese	118 (20.7)	43 (14.9)	75 (29.0)		25 (22.9)	93 (21.1)	
Body fat							
Underweight	48 (8.4)	29 (10.0)	19 (7.3)	0.023*	22 (20)	26 (5.9)	
Normal	198 (34.7)	117 (40.3)	80 (30.8)		49 (44.5)	149 (33.8)	< 0.001*
Overweight	130 (22.8)	66 (22.8)	64 (24.6)		20 (18.2)	110 (24.9)	
Obese	175 (30.6)	78 (26.9)	97 (37.3)		19 (17.3)	156 (35.4)	
Waist circumference							
Normal	171 (29.9)	95 (33.3)	76 (33.0)	0.976	81 (79.4)	90 (21.8)	< 0.001*
Overweight	107 (18.7)	60 (21.1)	47 (20.4)		9 (8.8)	98 (23.7)	
Obese	237 (41.5)	130 (45.6)	107 (46.5)		12 (11.8)	225 (54.5)	

Some variable measurements were missing and may therefore not sum up to 565. Chi square used to find differences by community and gender. *Significant at *p*-value < 0.05. Fisher Exact Test was used to ran the analysis for cell with counts less than 5

Table 3 shows the community and weight status of study participants by their physical activity category. Across all measures, with the exception of visceral fat, higher proportion of overweight and obese participants did not meet WHO recommendation for physical activity per week. A higher proportion of urban and female participants did not meet the WHO recommendation for physical activity.

**Table 3:** Community and weight status by physical activity category

Variable	Total	Not meeting WHO recommendation n(%)	Meeting WHO recommendation n(%)	*P*-value
BMI				
Underweight	9 (1.7)	5 (1.9)	4 (1.5)	
Normal	237 (43.9)	95 (35.8)	142 (51.6)	0.003
Overweight	167 (30.9)	91 (34.3)	76 (27.6)	
Obese	127 (23.5)	74 (27.9)	53 (19.3)	
Visceral fat				
Normal	419 (78.5)	187 (71.6)	232 (85.0)	< 0.001
Obese	115 (21.5)	74 (28.4)	41 (15.0)	
Body fat				
Underweight	46 (8.6)	17 (6.5)	29 (10.6)	
Normal	192 (35.9)	81 (31.0)	111 (40.5)	0.007
Overweight	125 (23.4)	63 (24.1)	62 (22.6)	
Obese	172 (32.1)	100 (38.3)	72 (26.3)	
Waist circumference				
Normal	166 (33.3)	62 (25.8)	104 (40.2)	
Overweight	104 (20.8)	48 (20.0)	56 (21.6)	0.001
Obese	229 (45.9)	130 (54.2)	99 (38.2)	
Community				
Rural	280 (51.1)	115 (42.6)	165 (59.4)	< 0.001
Urban	268 (48.9)	155 (57.5)	113 (40.6)	
Gender				
Male	111 (20.4)	43 (16.0)	68 (24.8)	0.010
Female	447 (79.8)	226 (84.0)	211 (75.6)	

WHO recommendation is equivalent to 600 or more of Metabolic equivalents per week. Chi-square test was used to find differences by community and gender. Fisher Exact Test was done for cells with counts less than 5

Tables 4 and 5 show principal component analysis of household food frequency. A total of four components were extracted for each community. The four components explained 32.4 and 30.9% of the total variance for rural and urban respectively. For the rural community the four components extracted were diverse diet pattern, vegetable convenience pattern, non-convenience pattern and fast food pattern. These contributed 15, 6.1, 5.5 and 5.4% of the 32.4% variance respectively. The diverse pattern consisted of foods from almost all the food groups. The vegetable pattern consisted of diet beverages, fresh and cooked vegetables, restaurant meals and nuts. The non-convenience pattern consisted of tea, coffee, breakfast cereal, sugar, nuts, cooked vegetables and low consumption of raw vegetables and ready to eat meals. The fast food pattern consisted of fried fish, fast foods, restaurant meals fruit and root tubers. For the urban community, the four components were diverse pattern, meat pattern, staple pattern and non-meat pattern. These explained 12.1%. 7.6, 5.9 and 5.4% of the total variance respectively. The diverse pattern as with the rural consisted of foods from almost all the food groups. The meat pattern consisted of ready to eat foods, organ meat, meat, fish, chicken, fruit and legumes. The staple pattern consisted of fried fish, maize, salted fish and raw vegetables and low likelihood for the consumption of ready to eat foods, processed meat and commercial bread. The non-meat pattern consisted of nuts, fish, cereal, legumes and salty snacks with low consumption of organ meat. Tables 6 and 7 show partial spearman correlation adjusted for age, gender and metabolic equivalents between the four components and body composition measures. Among urban participants component 3 (staple pattern) showed a negative correlation with BMI -0.163(0.029) and visceral fat − 0.186(0.013).

**Table 4:** Principal component analysis (PCA) of household food frequency (RURAL)

Food items in the PCA	Diverse diet	Vegetable convenience pattern	Non convenience pattern	Fast food pattern
Instant noodles	0.642			
Processed milk	0.568			
Salty snacks	0.556			
Sugar sweetened beverages	0.555			
Tea or coffee	0.531		0.379	
Meat	0.522			
Confectionery	0.504			
Eggs	0.474			
Commercial bread	0.455	−0.364		
Rice	0.433			
Vegetables raw fresh	0.424		−0.365	
breakfast cereal	0.42		0.322	
Organ meat	0.418			
chicken	0.417			
friedfish	0.415			0.318
sweets	0.404			
Margarine or butter	0.358			
Processed meat	0.34			
Legumes				
Fish				
Milk				
Salted dried fish				
Diet beverages		0.571		
Vegetables fried		0.504		
Fast food	0.356	0.500		0.331
Restaurant meals	0.380	0.469		0.361
Nuts		0.337	0.307	
Sugar	0.363		0.497	
Ready to eat meals		−0.377	−0.470	
Pasta	0.371		−0.310	−0.454
Vegetables (cooked)		0.434	0.320	−0.438
Fried potatoes	0.303		−0.367	−0.402
Fruit	0.325			0.376
Roots tubers				0.356

Principal component analysis of food frequency data. Kaiser-Meyer-Olkin Measure of Sampling Adequacy 0.740, Bartlett’s Test of Sphericity < 0.001

**Table 5:** Principal component analysis (PCA) of household food frequency (URBAN)

Food items in PCA	Diverse diet	Meat pattern	Staple pattern	Non-meat pattern
eggs	0.606			
sweets	0.587			
confectionery	0.573			
Processed milk	0.519			
Margarine or butter	0.492			
milk	0.431			
Sugar sweetened beverages	0.429			
sugar	0.412	−0.358		
Instant noodles	0.397			
Processed meat	0.377		−0.356	
Vegetables fried	0.368			
Fried potatoes	0.363			
Ready to eat meals		0.535	−0.341	
Tea or coffee	0.404	−0.534		
Vegetable cooked	0.335	−0.511		
Organ meat	0.317	0.455		
meat		0.455		−0.32
Fried fish		0.432	0.346	
chicken		0.357		
fruit		0.336		
rice				
maize			0.524	
Salted dried fish	0.333		0.501	
Commercial bread	0.341		−0.377	
Vegetables raw fresh			0.332	
Diet beverages				
Roots tubers				
nuts				0.498
fish				0.457
Breakfast cereal	0.337			0.389
Fast food	0.325			−0.38
legumes	0.305	0.302		0.309
Salty snacks				0.301

Principal component analysis of food frequency data. Kaiser-Meyer-Olkin Measure of Sampling Adequacy 0.651, Bartlett’s Test of Sphericity < 0.001

**Table 6:** Partial correlation between body measures and PCA (RURAL)

Variables	Component 1 Diverse diet r (*p*-value)	Component 2 Vegetable convenience pattern	Component 3 Non-convenience pattern r (*p*-value)	Component 3 Fast food pattern r (*p*-value)
visceral fat	0.091 (0.141)	− 0.076 (0.215)	− 0.091 (0.139)	0.043 (0.490)
body fat	0.823 (0.183)	−0.111 (0.072)	− 0.039 (0.522)	0.061 (0.319)
BMI	0.085 (0.166)	−0.052 (0.402)	−0.088 (0.152)	0.025 (0.684)
waist circumference	0.069 (0.266)	0.121 (0.050)	0.023 (0.715)	0.017 (0.782)

Spearman partial correlation of body measure and PCA. Controlled for age, gender and metabolic equivalent category

**Table 7:** Partial correlation between body measures and PCA (URBAN)

Variables	Component 1 Diverse diet r (*p*-value)	Component 2 Meat pattern	Component 3 Staple pattern r (*p*-value)	Component 4 Non meat pattern r(*p*-value)
visceral fat	0.077 (0.308)	0.092 (0.222)	−0.186 (0.013)*	−0.086 (0.254)
body fat	0.844 (0.266)	0.019 (0.799)	−0.075 (0.316)	−0.125 (0.095)
BMI	0.106 (0.159)	0.094 (0.209)	−0.163 (0.029)*	−0.105 (0.163)
waist circumference	0.078 (0.297)	0.037 (0.626)	−0.101 (0.180)	−0.020 (0.795)

Spearman partial correlation of PCA and body measures. Controlled for age, gender and metabolic equivalent category. *P-value < 0.05

Table 8 shows a partial spearman correlation adjusted for age and gender between waist circumference, BMI and body composition measures. All variables showed significant correlations but the strongest correlations were between visceral fat and BMI *r* = 0.905(*p* < 0.001), body fat and BMI *r* = 0.851 (*p* < 0.001) and BMI and waist circumference *r* = 0.845(*p* < 0.001).

**Table 8:** Partial correlation between body composition, waist circumference and BMI

Control Variables	Variables	visceral fat r (*p*-value)	body fat r (*p*-value)	BMI r (*p*-value)	waist circumference r (*p*-value)
Age in years, Gender	visceral fat		0.746(< 0.001)	0.905(< 0.001)	0.799(< 0.001)
	body fat	0.746(< 0.001)		0.851(< 0.001)	0.766(< 0.001)
	BMI	0.905(< 0.001)	0.851(< 0.001)		0.845(< 0.001)

Spearman partial correlation of body composition, waist circumference and BMI

Table 9 shows two models of multinomial logistic regression for risk factors of central obesity determined by waist circumference. Multicollinearity was checked and carbohydrate and fibre had strong correlations with energy intake and protein respectively and were therefore excluded from the model. In model 1 the obese group was set as reference for the outcome variable and urban and female were set as reference for the explanatory variables, community and gender respectively. Metabolic equivalent category was added to model 1 to generate model 2. In model 1 males had about 22 times odds of being normal compared to females at *p*-value < 0.001 while rural dwellers had odds of about 1.7 times of being normal compared to urban participants and this was significant at *p* < 0.05. The odds of energy intake were low but significant in both models. The predictability of gender for obesity persisted in model 2 but community showed no significant predictability. Participants not meeting WHO recommendation for physical activity had a significant reduced odds of being normal compared to those meeting the recommendation.

**Table 9:** Multinomial logistic regression of predictors of obesity

waist circumference category	Explanatory variables	Odds ratios
Model 1		
Normal^+^	**Energy**	0.999 (0.999–1.000)**
	**Protein**	1.008 (0.997–1.019)
	**Fat**	0.996 (0.987–1.005)
	**Sugar**	0.998 (0.992–1.004)
	**Community**	
	Rural	1.684 (1.039–2.729)*
	Urban^a^	
	**Gender**	
	Male	21.968 (10.876–44.373)***
	Female^a^	
Overweight	**Energy**	0.999 (0.999–1.000)**
	**Protein**	1.004 (0.994–1.015)
	**Fat**	0.999 (0.990–1.007)
	**Sugar**	1.002 (0.995–1.008)
	**Community**	
	Rural	1.198 (0.741–1.936)
	Urban^a^	
	**Gender**	
	Male	1.991 (0.790–5.018)
	Female^a^	
Model 2		
Normal^+^	**Energy**	0.999 (0.999–1.000)*
	**Protein**	1.008 (0.997–1.019)
	**Fat**	0.996 (0.987–1.005)
	**Sugar**	0.998 (0.992–1.004)
	**Community**	
	Rural	1.449 (0.881–2.385)
	Urban^a^	
	**Gender**	
	Male	19.715 (9.723–39.978)***
	Female^a^	
	**Metabolic equivalent category**	
	Not meeting WHO recommendation	0.585 (0.363–0.943)*
	Meeting WHO recommendation^a^	
Overweight	**Energy**	0.999 (0.999–1.000)*
	**Protein**	1.004 (0.994–1.016)
	**Fat**	0.999 (0.990–1.007)
	**Sugar**	1.001 (0.995–1.008)
	**Community**	
	**Rural**	1.109 (0.675–1.823)
	**Urban**^**a**^	
	**Gender**	
	Male	1.903 (0.753–4.810)
	Female^a^	
	**Metabolic equivalent category**	
	Not meeting WHO recommendation	0.729 (0.449–1.182)
	Meeting WHO recommendation^a^	

Multinomial logistic regression of the predictors of obesity

model 1 *p < 0.05, **p < 0.01, ***p < 0.001. ^a^Variable set as reference. +Reference for weight category is obesity. Cox and Snell R-Squared is 0.243 and Nagelkerke R-Squared is 0.277

model 2 *p < 0.05, **p < 0.01, ***p < 0.001. ^a^Variable set as reference. +Reference for weight category is obesity. Cox and Snell R-Squared is 0.248 and Nagelkerke R-Squared is 0.283

## Discussion

This study evaluated the association between nutrient consumption, physical activity and body composition of rural and urban adults in Ghana. The study had a higher number of female participants compared to males and this was because it was mostly females who were met at the households. This finding is comparable to reports from the 2014 Ghana Demographic and Health survey [22]. The prevalence of overweight and obesity using BMI was 29.9 and 22.9% respectively with a total prevalence of 52.8%. The prevalence of overweight and obesity found in this study is comparable to what has been reported by some studies even though others have reported slightly higher or lower prevalence [22–25]. Several studies have reported a significantly higher prevalence of obesity by BMI among urban compared to rural dwellers but in this study, no significant difference was found [23, 26]. This implies that rural communities are gradually catching up with urban communities and there is therefore the need for further investigations and interventions to halt exponential positive BMI changes in rural settings. Bixby et al., reviewed population-based studies from 1985 to 2017 and they found that global increase in BMI and obesity is driven by increases in BMI among rural residents [27]. This finding together with findings from this study implies the need for an integrated approach to address the problem of malnutrition among rural residents. The problem of undernutrition in rural areas which has been the focus over the years seem to give way to overweight and obesity driven by increased consumption of cheap caloric dense foods. Traditional outlets and small shops in rural areas are adapting their sales to incorporate ultra-processed foods. The nutrition transition is no longer a phenomenon pertaining only to urban areas but to rural communities as well. Total energy and carbohydrate intake were significantly higher among rural compared to urban participants. Though not statistically significant, sugar and saturated fat intake was higher among the urban sample. This finding supports the leading role cities play in the nutrition transition through higher availability and consumption of processed foods with corresponding low intakes of staple foods [28]. Additionally, multinomial logistic regression results from this study indicates that rural communities are associated with normal weight compared to urban centres but this was not significant when metabolic equivalent category (physical activity) was included in the model. This finding shows the importance of physical activity in the maintenance of weight irrespective of geographical location. Provision of environments that support healthful food consumption and physical activity has potential to reduce obesity in both rural and urban areas. Availability of sugary foods notably sugar-sweetened beverages in urban communities coupled with low levels of physical activity are associated with weight gain and risk of type 2 diabetes. Central obesity determined by visceral fat was higher among urban participants; central obesity is highly associated with cardiovascular diseases and poses a higher cardiometabolic risk compared to generalized obesity [18, 19].

Prevalence of obesity determined by BMI, waist circumference and body fat were higher among females compared to males. Most studies have also reported a higher prevalence of obesity among women compared to men though most of these studies have only used BMI [29, 30]. BMI does not have gender specific cut offs but in this study where visceral fat, total body fat and waist circumference were used (all of which have gender specific cut offs), prevalence across all determinants was significantly higher for females compared to males with the exception of visceral fat that showed no significant difference. Several factors put women at higher risk of obesity compared to men. Sedentary behavior such as long hours watching television and parity have been documented to cause weight gain in women [31]. Even though pregnancy and childbirth are associated with weight gain and retention it is possible for women to lose almost all the weight gained during pregnancy and child birth [32, 33]. This calls for post-natal education and interventions to empower women to avoid obesogenic behaviors post-partum which are thought to aid in lactation but are rather fattening. High occurrence of obesity among women is related to adverse pregnancy outcomes such as pre-mature delivery and low birth weight babies. Low birth weight is a risk factor for obesity in adulthood and this trend has led to developing countries experiencing a double burden of disease [33]. The prevalence of central obesity was also higher among females and this puts them at risk of chronic non-communicable diseases. High amount of total body fat is associated with elevated blood pressure and this was higher among women compared to men [31]. Multinomial logistic regression also revealed that female gender poses a significant risk to the development of obesity and this was irrespective of metabolic equivalent category. Several interventions have been tailored toward socio-economic empowerment of women in Ghana but findings from this study calls for more sensitive interventions to be implemented to address the health challenges of women and also empower women toward taking charge of their health in order to achieve optimal health status.

The WHO recommends achieving minimum of 600 metabolic equivalents in a week which translates to 150 min of moderate intensity exercise or 75 min of vigorous intensity activity as a way to improve and maintain a healthy weight and cardiometabolic state. Approximately half of study participants majority of whom were females did not meet WHO recommendation for physical activity. Participants not achieving this recommendation had a higher prevalence of obesity across most of the measures. There is the need for public education on the importance of physical activity in achieving and maintaining a healthy weight and cardio metabolic health.

Fat, protein, sugar and carbohydrate contribute to total energy intake and caloric intake positively associates with BMI. Principal component analysis revealed four different patterns for the two communities. For the urban community, the staple pattern (pattern 3) showed an inverse significant relationship with visceral fat and BMI. Pattern 3 comprised of staple foods like maize, fried and salted fish and raw vegetables and low consumption of commercial bread, ready to eat meals and processed meat. Commercial bread, ready to eat meals and processed meat consumption are associated with increased body mass index and incidence of obesity while vegetable intake is associated with lower body mass index. In this study the protective effect persisted even after adjusting for age, gender and physical activity status [30]. This implies that an adaptation of a healthy diet pattern is instrumental in curbing the rise in obesity. These findings support the call by the WHO for countries to provide a healthy food environment through the regulation of ultra-processed foods as means to halt the obesity pandemic. Among rural dwellers, none of the patterns generated significantly associated with any of the obesity measures.

Waist circumference is a reliable tool for determining abdominal obesity. In this study it predicted the highest prevalence of overweight and obesity and this implies that BMI is often associated with the underestimation rather than the overestimation of adiposity among populations. Its strong positive correlation with other body composition measures supports the reliability of waist circumference as a measure of obesity and adiposity. This simple measurement can be used alone as a diagnostic tool to assess obesity and adiposity among populations and in clinical practice.

## Conclusion

This study indicates that overweight and obesity continue to rise in Ghana, especially among females. Prevalence of obesity determined by BMI was similar among rural and urban dwellers. Interventions that regulate the food environment and makes affordable and available healthy food options such as vegetables are needed to control the rise in obesity rate and prevalence.

### Limitation

The higher number of females compared to males is a limitation to this study and may have led to biases in the obesity and physical activity difference reported along gender lines.

## Data Availability

The data analyzed for this manuscript are available from the corresponding author and can be made accessible upon reasonable request.
